# Bioinformatics analysis identifies COL1A1, THBS2 and SPP1 as potential predictors of patient prognosis and immunotherapy response in gastric cancer

**DOI:** 10.1042/BSR20202564

**Published:** 2021-01-08

**Authors:** Yali Wang, Kun Zheng, Xiuqiong Chen, Rui Chen, Yanmei Zou

**Affiliations:** Department of Oncology, Tongji Hospital, Tongji Medical College, Huazhong University of Science and Technology, Wuhan, Hubei, China

**Keywords:** Bioinformatics, gastric cancer, hub genes, immunotherapy

## Abstract

**Background:** The present study aimed to use bioinformatics tools to explore pivotal genes associated with the occurrence of gastric cancer (GC) and assess their prognostic significance, and link with clinicopathological parameters. We also investigated the predictive role of COL1A1, THBS2, and SPP1 in immunotherapy.

**Materials and methods:** We identified differential genes (DEGs) that were up- and down-regulated in the three datasets (GSE26942, GSE13911, and GSE118916) and created protein–protein interaction (PPI) networks from the overlapping DEGs. We then investigated the potential functions of the hub genes in cancer prognosis using PPI networks, and explored the influence of such genes in the immune environment.

**Results:** Overall, 268 overlapping DEGs were identified, of which 230 were up-regulated and 38 were down-regulated. CytoHubba selected the top ten hub genes, which included SPP1, TIMP1, SERPINE1, MMP3, COL1A1, BGN, THBS2, CDH2, CXCL8, and THY1. With the exception of SPP1, survival analysis using the Kaplan–Meier database showed that the levels of expression of these genes were associated with overall survival. Genes in the most dominant module explored by MCODE, COL1A1, THBS2, and SPP1, were primarily enriched for two KEGG pathways. Further analysis showed that all three genes could influence clinicopathological parameters and immune microenvironment, and there was a significant correlation between COL1A1, THBS2, SPP1, and PD-L1 expression, thus indicating a potential predictive role for GC response to immunotherapy.

**Conclusion:** ECM–receptor interactions and focal adhesion pathways are of great significance in the progression of GC. COL1A1, THBS2, and SPP1 may help predict immunotherapy response in GC patients.

## Introduction

Gastric cancer (GC) is the fourth most common human malignant tumor worldwide and the second leading cause of cancer death worldwide [1]. It has an insidious onset and is mostly detected at an intermediate to advanced stage [2]. Therefore, even though multiple therapies such as surgery, chemotherapy, radiation therapy, and targeted therapies alone or in combination have played a role in the treatment of GC, the overall 5-year survival rate is still less than 20% [3]. Such low survival rate is mainly due to the heterogeneity of GC [4]. Genetic mutations are known to play a key role in the development, progression, and prognosis of a variety of diseases, and genetic biomarkers have been widely used in the diagnosis and targeted treatment of various diseases [5–7]. In this context, identifying the mutated genes in GC and developing effective therapeutic strategies is a crucial factor in improving patient prognosis.

In recent years, the analysis of biological information, also known as bioinformatics, has attracted a great deal of attention and sustained breakthroughs in the search for oncogenic genes. Various functions for molecular typing, prognostic prediction, new targeted drug development applications and biomarkers of prognosis have been confirmed [8–10]. Thus, we used bioinformatics to identify genes predictive of GC prognosis. In the present study, we analyzed three mRNA gene chip datasets (GSE26942, GSE13911, and GSE118916), and identified overlapping DEGs between tumor and normal samples. The prognostic value of the identified genes was assessed by survival analysis. The efficacy of immune checkpoint inhibitors was influenced by a combination of factors such as tumor genomics, in vivo PD-L1 levels, and characteristics of the tumor microenvironment. Therefore, we selected core genes to explore their effects on the immune microenvironment and PD-L1 expression in GC. The genes identified in the present study may be potential prognostic biomarkers and therapeutic targets for GC. Further, the association of these genes with the GC immune environment could be further explore.

## Materials and methods

### Microarray data

To obtain gene expression datasets for GC in this study, we downloaded three datasets from the GEO database (http://www.ncbi.nlm.nih.gov/geo/): GSE26942, GSE13911, and GSE118916. These RNA profiles were based on GPL6947 (Illumina HumanHT-12 V3.0 expression bead chip), GPL570 platform (Affymetrix Human Genome U113 Plus 2.0 array), and GPL15207 (Affymetrix Human Gene Expression Array), and contained 258 GC tissues and 58 normal tissues. The GSE26942 profile contains 205 GC tissues and 12 normal tissues, the GSE13911 profile 38 GC tissues and 31 normal tissues, and the GSE118916 profile 15 GC tissues and 15 normal tissues.

### DEGs screening

We obtained and exclusively selected differential genes (DEGs) by GEO2R (http://www.ncbi.nlm.nih.gov/geo/geo2r/) analysis from the GEO database. Genes with *P* value <0.05 and | logFC | > 1 were considered as DEGs in this dataset. A Venn diagram was used to visualize overlapping DEGs between the above datasets.

### Function and pathway enrichment analysis

Overlapping DEGs were used to map possible biological functions, and gene ontology (GO) enrichment analysis was performed in terms of biological processes (BP), cellular components (CC), and molecular function (MF) [11,12]. Kyoto Encyclopedia of Genes and Genomes (KEGG) pathway enrichment analysis was then performed to investigate potential enrichment for signaling pathways among the overlapping DEGs

The KEGG database is widely used to explore information about biological pathways, genomes, diseases, chemicals, and to identify functional and metabolic pathways [13]. GO and KEGG pathway enrichment analysis were performed using the DAVID website. Bubble charts were created using R language.

### Establishment of the PPI network, modules selection, and identification of hub genes

To investigate the protein interactions of DEGs, we submitted them to the Interaction Gene Search tool (STRING) (http://string.embl.de/) [14]. We then used Cytoscape software to integrate and visualize the protein–protein interaction (PPI) network. Molecular complex detection (MCODE) was applied to screen the modules of the PPI network, and the core modules were selected. In addition, hub genes in the network were identified using the cytoHubba application in Cytoscape software. The maximum group centrality of each gene in the network was calculated by the maximal clique centrality (MCC) score. We regarded the top 10 genes as hub genes.

### Relationship between hub genes and survival analysis

We explored the main functions and pathway enrichment analysis of the hub genes by searching the DAVID website (https://david.ncifcrf.gov/) [15]. To further validate the reliability of the hub genes, their expression was first examined using the Gene Expression Profiling Interaction Analysis (GEPIA) database analysis [16]. The relationship between hub genes and patient prognosis was then analyzed by Kaplan-Meier curves (http://kmplot.com/) [17], and *P*<0.05 was considered to be statistically significant.

### Expression and mutation analysis of hub genes

The relationship between gene expression and mutations of hub genes was carried out using the online c-BioPortal database (http://cbioportal.org) [18]. A total of 478 patients/samples were selected for further analysis. OncoPrint was obtained using an online database at c-BioPortal.

### Link between Hub genes expression levels and GC clinicopathological parameters

ONCOMINE (https://www.oncomine.org/) [19] was used to explore the gene expression levels of potential biomarkers in GC tumor tissue compared with normal tissue. UALCAN (http://ualcan.path.uab.edu/) [20], an online database for the analysis and mining of cancer data, was used to explore the relationship between mRNA expression levels of potential biomarkers and the clinicopathological parameters of GC patients. *P* value < 0.05 was considered statistically significant. Immunohistochemistry was performed using the Human Protein Atlas (THPA) (http://www.proteinatlas.org/) [21]. We evaluated the expression levels of hub genes between normal gastric tissues and GC tissues from THPA.

### Relationship between hub genes and immune infiltrates

The correlation between hub genes and immune infiltration (B cells, CD4+ T cells, CD8+ T cells, neutrophils, macrophages, and dendritic cells) in The Cancer Genome Atlas (TCGA) of Stomach Adenocarcinoma (STAD) was carried out using the Tumor Immune Estimation Resource (TIMER) platform [22].

### The expression between hub genes and PD-L1 gene expression

The relationship between each of the three hub genes and PD-L1 gene expression in the clinical data of 580 GC patients in TCGA database are available from UCSC (https://genome-cancer.ucsc.edu/).

## Results

### Identification of DEGs

**Figure 1:**
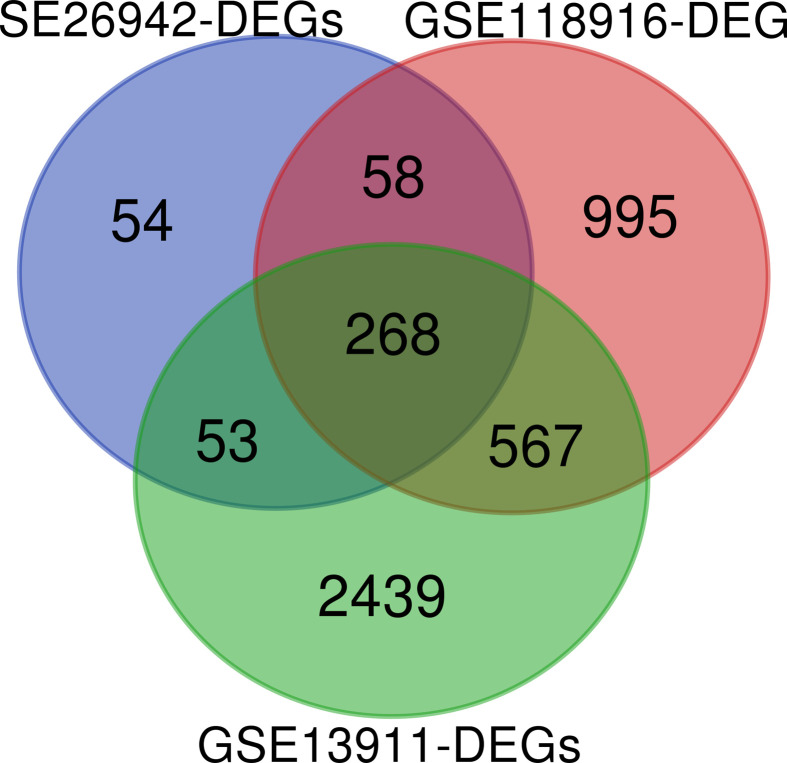
A Venn diagram of DEGs A comparison between three datasets with 433, 3327, and 1888 DEGs, revealed 268 common DEGs between GC and normal tissues.

After a comprehensive analysis of the three microarray datasets, we identified 268 overlapping DEGs, of which 230 genes were up-regulated and 38 genes were down-regulated. A Venn diagram for the three datasets is shown in Figure 1.

### GO and KEGG pathway enrichment analyses of DEGs

**Figure 2:**
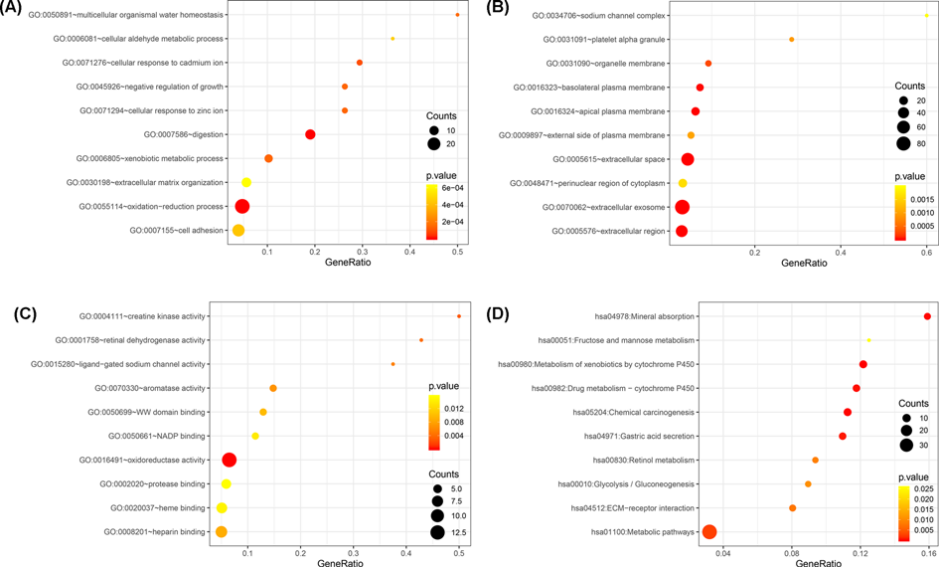
Functional enrichment analysis and KEGG pathway analysis of DEGs (**A**) Functional enrichment analysis of BP; (**B**) Functional enrichment analysis of CC; (**C**) Functional enrichment analysis of MF; (**D**) KEGG pathway analysis.

GO and KEGG pathway enrichment analyses were performed using the DAVID website. The top 10 terms of BP, CC, MF, and KEGG pathways are shown in Figure 2. For biological processes, results from the GO analysis revealed an enrichment for genes linked to processes such as digestion, oxidation–reduction process, cellular response to cadmium ion, xenobiotic metabolic process, cellular response to zinc ion, negative regulation of growth, multicellular organismal water homeostasis, cell adhesion, cellular aldehyde metabolic process, and extracellular matrix organization (Figure 2A). Cellular component analysis showed that the DEGs were particularly enriched for categories such as extracellular space, extracellular exosome, apical plasma membrane, extracellular region, basolateral plasma membrane, organelle membrane, platelet alpha granule, external side of plasma membrane, perinuclear region of cytoplasm, and sodium channel complex (Figure 2B). Regarding molecular functions, analysis of DEGs revealed a significantly enrichment for functions such as oxidoreductase activity, creatine kinase activity, retinal dehydrogenase activity, ligand-gated sodium channel activity, aromatase activity, heparin binding, WW domain binding, NADP binding, heme binding, and protease binding (Figure 2C). Additionally, the results of KEGG pathway analysis revealed that the DEGs were mostly linked to the metabolism of xenobiotics by cytochrome P450, chemical carcinogenesis, mineral absorption, drug metabolism – cytochrome P450, gastric acid secretion, mineral absorption, metabolic pathways, ECM-receptor interaction, retinol metabolism, glycolysis/gluconeogenesis, fructose, and mannose metabolism (Figure 2D).

### Construction of PPI network and identification of hub genes

**Figure 3:**
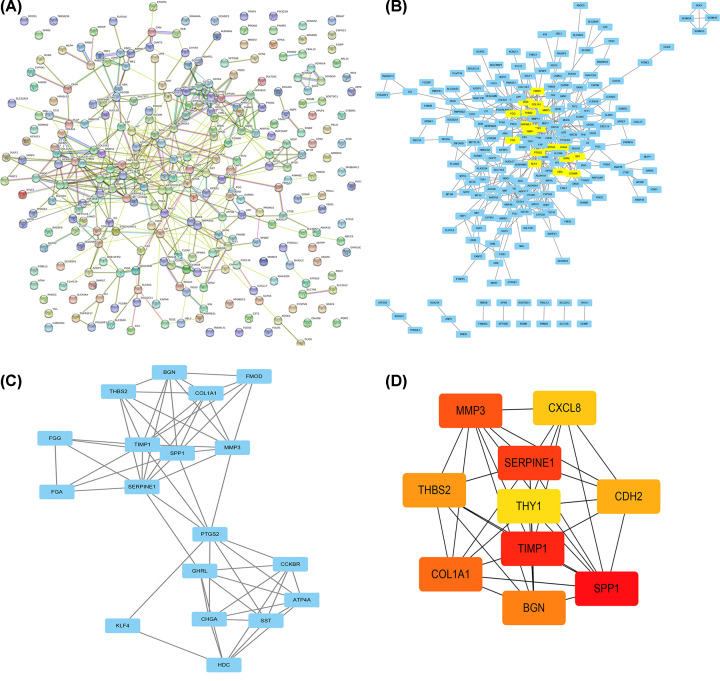
Selection of hub genes (**A**) PPI network by STRING analysis; (**B**) PPI network was produced using Cytoscope Software; (**C**) Core modules were designed using MCODE; (**D**) Top 10 genes network. The top 10 genes of the MMC method were chosen using the CytoHubba plug-in. The more forward ranking is represented by a redder color.

A PPI network from the STRING database was established to predict the potential interactions of the DEGs at the protein level (Figure 3A). By opening the PPI network from the STRING database with Cytoscope software, we constructed a co-expression network consisting of 213 nodes and 489 edges (Figure 3B). MCODE was applied to screen modules of the PPI network. The most pivotal module in the PPI network was selected (Figure 3C). In addition, hub genes in the network were identified using the cytoHubba application in Cytoscape software. The top 10 genes were selected as hub genes, including SPP1, TIMP1, SERPINE1, MMP3, COL1A1, BGN, THBS2, CDH2, CXCL8, and THY1 (Figure 3D).

### Functional analysis of hub genes and survival analysis

**Figure 4:**
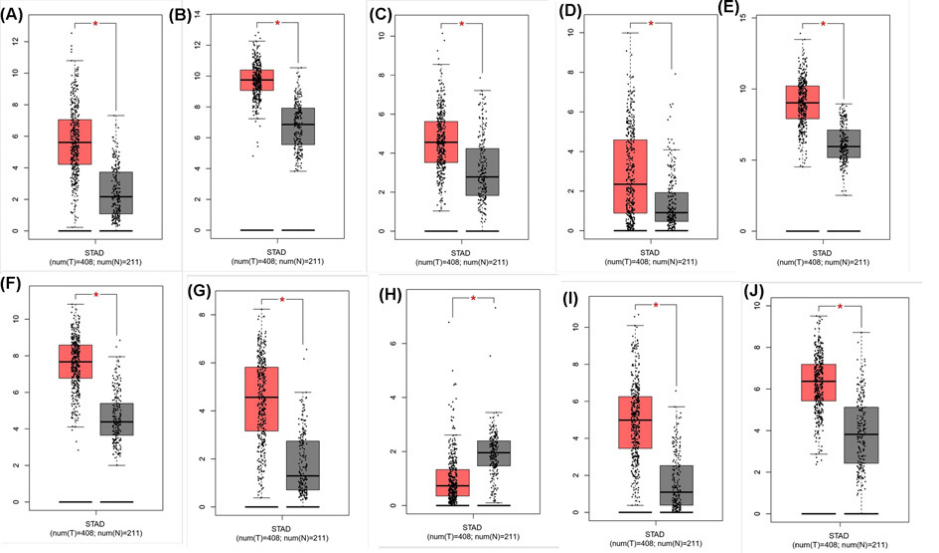
Expression of hub genes in normal and GC tissues (**A**) SPP1; (**B**) TIMP1; (**C**) SERPINE1; (**D**) MMP3; (**E**) COL1A1; (**F**) BGN; (**G**) THBS2; (**H**) CDH2; (**I**) CXCL8; (**J**) THY1.

**Figure 5:**
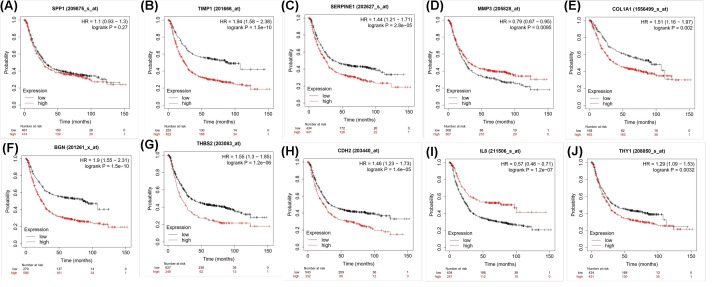
Relationship between the expression of hub genes and overall survival estimated using the Kaplan–Meier plotter (**A**) SPP1; (**B**) TIMP1; (**C**) SERPINE1; (**D**) MMP3; (**E**) COL1A1; (**F**) BGN; (**G**) THBS2; (**H**) CDH2; (**I**) CXCL8; (**J**) THY1.

Most hub genes were involved in the core module. Enrichment analysis using the DAVID website revealed that hub genes were mainly enriched for the following GO terms: extracellular region, cell adhesion, extracellular matrix organization, extracellular space, extracellular matrix, and extracellular matrix disassembly (Table 1). A *P*-value <0.05 was set as the cut-off value. KEGG results included ECM–receptor interaction and focal adhesion (Table 1). Based on the GEPIA website, all hub genes were differentially expressed between gastric tumor tissues and normal tissues (Figure 4), which further indicated that these genes may contribute to the occurrence and development of GC. The Kaplan–Meier plotter database confirmed that higher expression levels of these genes were related to lower overall survival, except for SPP1, MMP3, and CXCL8. In fact, the mRNA expression of SPP1 was independent of overall survival. Higher levels of MMP3 and CXCL8 expression had positive impacts on overall survival (Figure 5).

**Table 1:** Functional enrichment analysis and KEGG pathway analysis of hub genes

Category	Term	Count	*P* value
GOTERM_BP_DIRECT	GO:0007155∼cell adhesion	5	6.23E-05
GOTERM_BP_DIRECT	GO:0030198∼extracellular matrix organization	4	1.25E-04
GOTERM_BP_DIRECT	GO:0022617∼extracellular matrix disassembly	3	7.13E-04
GOTERM_BP_DIRECT	GO:0001525∼angiogenesis	3	0.00594
GOTERM_BP_DIRECT	GO:0044344∼cellular response to fibroblast growth factor stimulus	2	0.01597
GOTERM_BP_DIRECT	GO:0043434∼response to peptide hormone	2	0.02334
GOTERM_BP_DIRECT	GO:0030574∼collagen catabolic process	2	0.03379
GOTERM_BP_DIRECT	GO:0030336∼negative regulation of cell migration	2	0.04979
GOTERM_CC_DIRECT	GO:0005576∼extracellular region	8	1.27E-06
GOTERM_CC_DIRECT	GO:0005615∼extracellular space	6	2.14E-04
GOTERM_CC_DIRECT	GO:0031012∼extracellular matrix	4	3.31E-04
GOTERM_CC_DIRECT	GO:0070062∼extracellular exosome	6	0.00636
GOTERM_CC_DIRECT	GO:0005578∼proteinaceous extracellular matrix	3	0.00725
GOTERM_CC_DIRECT	GO:0031093∼platelet alpha granule lumen	2	0.02684
GOTERM_CC_DIRECT	GO:0005604∼basement membrane	2	0.03835
GOTERM_CC_DIRECT	GO:0005581∼collagen trimer	2	0.04454
GOTERM_MF_DIRECT	GO:0050840∼extracellular matrix binding	2	0.01378
GOTERM_MF_DIRECT	GO:0005515∼protein binding	9	0.02599
GOTERM_MF_DIRECT	GO:0005201∼extracellular matrix structural constituent	2	0.03517
KEGG_PATHWAY	hsa04512:ECM–receptor interaction	3	0.00421
KEGG_PATHWAY	hsa04510:Focal adhesion	3	0.02219

### Mutational analysis of hub genes

**Figure 6:**
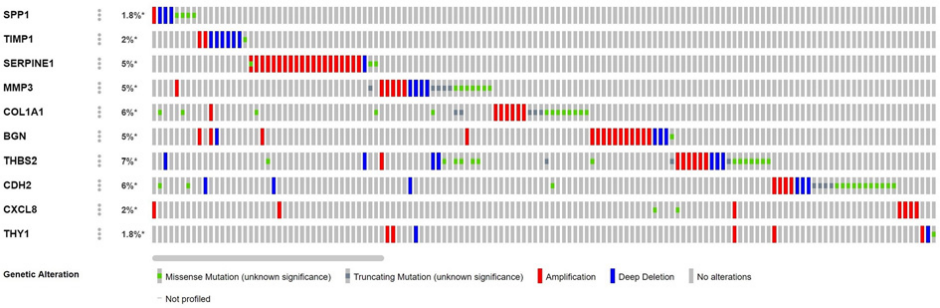
Genetic mutation analysis of 10 hub genes in GC The mutation rate is 7% for THBS2; 6% for COL1A1 and CDH2; 5% for SERPINE1, MMP3, and BGN; and less than 5% for SPP1, CXCL8, TIMP1, and THY1.

In the mutational analysis of the hub genes, we found that mutations were mainly present in THBS2, COL1A1, and CDH2 (Figure 6). Among all mutation types, amplification was the most common.

### Correlation between hub genes expression levels and GC clinicopathological parameters

**Figure 7:**
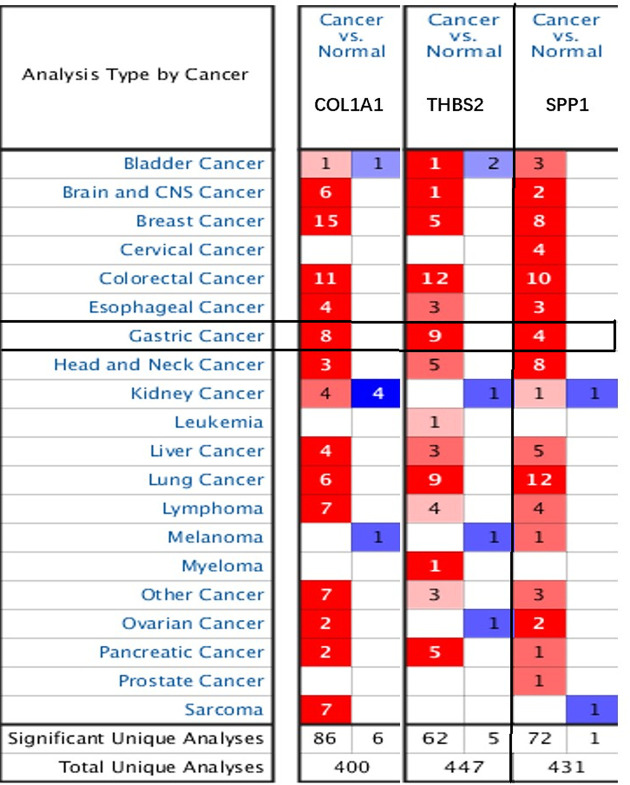
mRNA transcription levels of potential GC biomarkers using ONCOMINE Numbers in each cell indicate the number of studies that met the target genes screening criteria. The depth of color indicates the level of gene expression, with red indicating overexpression and blue indicating down-regulation.

**Figure 8:**
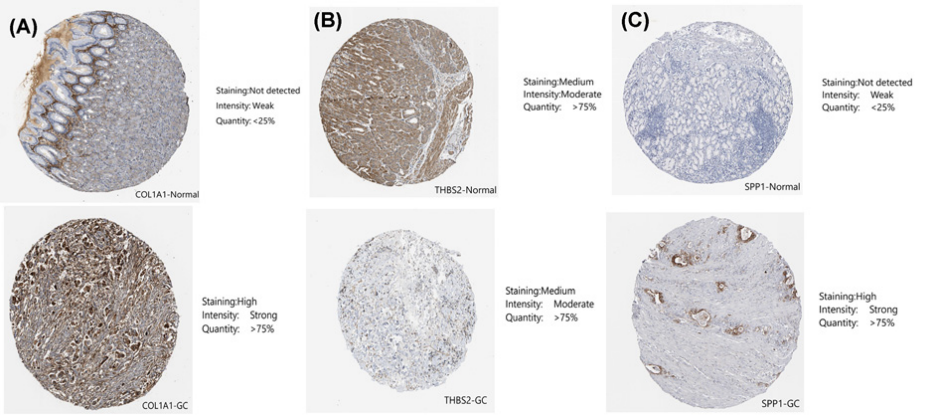
Immunohistochemistry from THPA was used to explore differences in the gene expression of three genes between normal and GC tissues (**A**) COL1A1; (**B**)THBS2; (**C**) SPP1.

**Figure 9:**
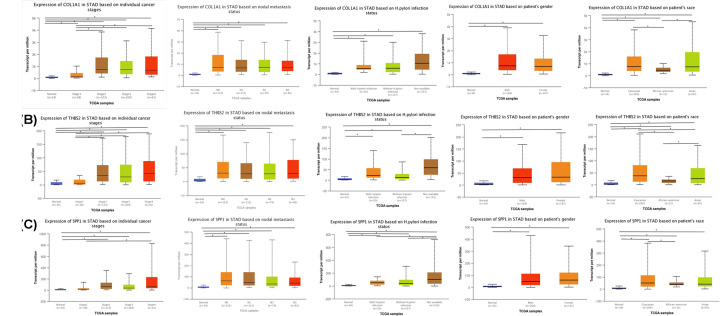
Relationship between clinicopathological parameters and hub genes (**A**) Clinicopathological parameters of COL1A1; (**B**) Clinicopathological parameters of THBS2; (**C**) Clinicopathological parameters of SPP1.

COL1A1, THBS2, and SPP1 were found to participate in both ECM-receptor interaction and focal adhesion (Table 1). Furthermore, we used ONCOMINE to explore the expression levels of potential biomarkers in GC tumor tissue compared with normal tissue. We found 400, 447, and 431 studies on COL1A1, THBS2, and SPP1, respectively (Figure 7). To further validate their prognostic significance of the three genes, we looked at their levels of expression in normal gastric tissues compared to GC tissues in THPA. Immunohistochemistry indicated that the expression levels of COL1A1 and SPP1 are up-regulated in GC tissues. THBS2 protein did not show significant differences in immunohistochemistry between GC and normal gastric tissues (Figure 8). We further explored the relationships between COL1A1, THBS2, and SPP1 expression levels and clinicopathological parameters of GC patients using UALCAN analysis. As shown in Figure 9A–C, the mRNA transcription levels of the three genes, considered potential biomarkers of GC, were positively correlated with individual cancer stages, and nodal metastasis status in GC, but independent of *Helicobacter pylori* infection and sex. Notably, the levels of expression of the three genes differed significantly among different human populations (Figure 9).

### Correlation between hub genes and immune infiltrates

**Figure 10:**
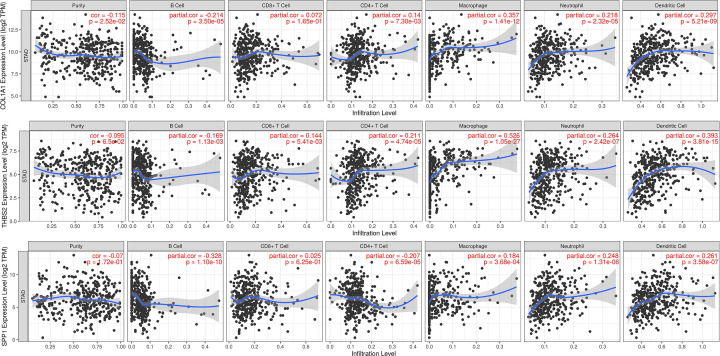
Correlation analysis between immune infiltrates and expression of COL1A1, THBS2, and SPP1 genes The expression levels of COL1A1 showed a significant positive correlation with macrophages and CD4 + T cells, and it was a definite negative correlation with B cells. THBS2 was highly correlated with macrophages and dendritic cells. SPP1 showed the most significant negative correlation with B cells, and no correlation with CD8 + T cells. With the exception of CD4 + T cells, SPP1 showed a certain degree of positive correlation with the remaining immune infiltrating cells.

The efficacy of immune checkpoint inhibitors is influenced by a combination of factors such as tumor genomics, *in vivo* PD-L1 levels, and characteristics of the tumor microenvironment. To provide further insight into the management of immunotherapy in GC, the present study analyzed the correlation between hub genes and tumor immune infiltration via the TIMER platform (Figure 10). The expression levels of COL1A1 showed a significant positive correlation with macrophages and CD4 + T cells (cor = 0.357, *P*<0.0001), and a negative correlation with B cells (cor = −0.214, *P*<0.0001). THBS2 was highly correlated with macrophages (cor = 0.526, *P*<0.0001) and dendritic cells (cor = 0.393, *P*<0.0001). In addition, it also had some correlation with CD8+ T cells and CD4 + T cell expression (*P*<0.005). SPP1 showed the most significant negative correlation with B cells (cor = −0.328, *P*<0.0001), and no correlation with CD8 + T cells (*P*>0.05). With the exception of CD4 + T cells, SPP1 showed a certain degree of positive correlation with the remaining immune infiltrating cells.

### Association between hub genes and PD-L1 gene expression in GC patients

**Figure 11:**
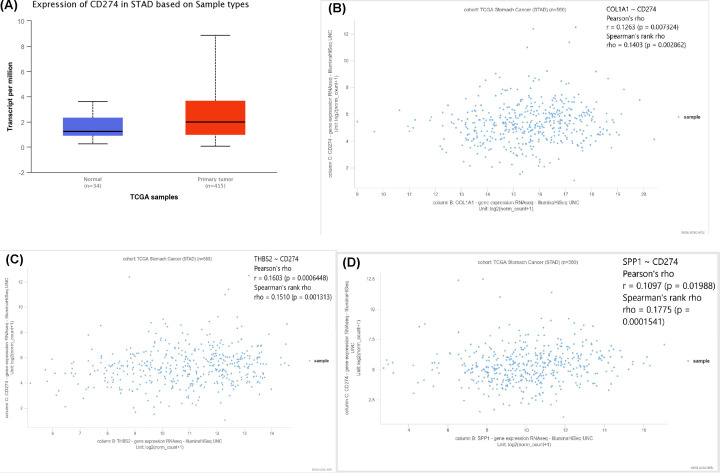
Correlation between expression of hub genes and PD-L1 expression (**A**) Expression of PD-L1 in normal versus GC tissues. (**B**) Co-identification of PD-L1 and COL1A1 in GC tissues. (**C**) Co-identification of PD-L1 and THBS2 in GC tissues. (**D**) Co-identification of PD-L1 and SPP1 in GC tissues.

After reviewing the literature, we chose to explore the association between gene mutations and PD-L1 gene expression to assess gene signature and immune relevance. Results from the UALCAN database showed a significant increase in PD-L1 expression in GC (Figure 11A) and that the three hub genes exist in the COL1A1/PD-L1 axis, THBS2/PD-L1 axis, and SPP1/PD-L1 axis (Figure 11B,C).

## Discussion

In recent years, immunotherapy has been extensively studied as a promising strategy for the treatment of cancer [23]. Programmed cell death ligand 1 (PD-L1) is an important immunomodulatory molecule that is highly expressed in many human cancer types [24,25], and can inhibit physiological and pathological pathways by interacting with its receptor PD-1 [26]. Despite the success of immune checkpoint therapy in melanoma and lung cancer, the efficacy of PD-1 or PD-L1 blockade in GC is not good. Clinically, the overall remission rate in GC patients treated with PD-1/PD-L1 inhibitors is only approximately 20% [27]. Further, PD- L1 is not an ideal biomarker for gastroesophageal cancer. The efficacy of immune checkpoint inhibitor single agents is influenced by tumor genomics, *in vivo* PD-L1 levels, and the tumor microenvironment [27–29]. For example, in GC, microsatellite instability and EBV status are predictive of the efficacy of immunosuppressive drugs [30,31]. Similarly, there are many factors related to the immune microenvironment that also predict whether a patient will respond to single-agent PD-1 inhibition. Such factors include high tumor antigen load, dendritic cells, CD4+T cell infiltration, CD8+T cell infiltration, and pro-inflammatory cytokines, among others [32–35]. The most well-known cytokine responsible for PD-L1 upregulation in the gastrointestinal tract is the regulatory cytokine interleukin 10 (IL10) [36]. In addition, single-agent effects may also be associated with altered PD-L1 expression due to genetic alterations and activation of the oncogenic signaling pathways PI3K/AKT, ERK, and JAK/STAT3 pathways [28,29].

An increasing number of reports have revealed oncogenic genes and other genetic marker relationships in GC. In the context of precision medicine, tailoring treatments based on a patient’s genotype is becoming increasingly important. Therefore, we selected hub genes to explore the assessment of gene signatures and their correlations with the tumor immunoenvironment.

Our results showed that the hub genes COL1A1, THBS2, and SPP1 were also able to alter cellular components in the tumor microenvironment, and that they had an axial relationship with PD-L1 expression. COL1A1, which encodes the most abundant protein of the collagen family, is a major component of the extracellular matrix and can influence cell behavior and tissue structure [37]. COL1A1 is considered to effectively suppress gene expression and inhibit the proliferation, migration, and invasion of GC cells [38]. THBS2 was identified as a potent inhibitor of angiogenesis and tumor growth, while promoting cell adhesion and migration [39]. THBS2 also affects the proteolysis of tumor cytoplasm, thereby contributing to certain proteins in the PI3K/AKT signaling pathway. Previous studies have observed that the expression of TGF-β, COL1A1, and THBS2 in GC cells is associated with the survival of GC patients in a time-dependent manner [40], suggesting that COL1A1 and THBS2 may affect PI3K by up-regulating the TGF-β signaling pathway. COL1A1 has also been observed to play a regulatory role in the JAK pathway [41]. Interaction of SPP1 and its receptor CD47 further inhibits angiogenesis by antagonizing nitric oxide signaling in endothelial and vascular smooth muscle cells which, in turn, affects the tumor microenvironment [42]. SPP1 can directly regulate interleukin 6 [43]. SPP1 is also associated with the expression of genes related to the PI3K/ AKT pathway and epithelial–mesenchymal transition (EMT) [44]. We explored that COL1A1, THBS2, and SPP1 were mainly involved in ECM receptor interactions and adhesion plaque pathways using KEGG pathway analysis. Further, they affect the immune microenvironment and upregulate the expression of PD-L1 through PI3K/AKT signaling pathway, JAK signaling pathway, and TGF-β signaling pathway. In addition, it is possible that more basic experiments are needed to elucidate the mechanism.

## Data Availability

The authors certify that all the original data in this research could be obtained from public database. The clinical data of GC patients was obtained from the TCGA database (https://tcga-data.nci.nih.gov/tcga/). The data of training groups are available at NCBI GEO (GSE26942 https://www.ncbi.nlm.nih.gov/geo/query/acc.cgi?acc=GSE26942, GSE13911 https://www.ncbi.nlm.nih.gov/geo/query/acc.cgi?acc=GSE13911, and GSE118916 https://www.ncbi.nlm.nih.gov/geo/query/acc.cgi?acc=GSE26942). All data generated or analyzed during the present study are included in this published article and its supplementary file information files.

## Abbreviations

BP: biological processes
CC: cellular components
DEGs: differential genes
EMT: epithelial–mesenchymal transition
GC: gastric cancer
GEPIA: Gene Expression Profiling Interaction Analysis
GO: Gene ontology
IL10: interleukin 10
KEGG: Kyoto Encyclopedia of Genes and Genomes
MCC: maximal clique centrality
MCODE: molecular complex detection
MF: molecular function
PD-L1: programmed cell death ligand 1
PPI: protein–protein interaction
STAD: stomach adenocarcinoma
TCGA: The Cancer Genome Atlas
TIMER: The Tumor Immune Estimation Resource
